# Does the Use of Different Indicators to Benchmark Antimicrobial Use Affect Farm Ranking?

**DOI:** 10.3389/fvets.2020.558793

**Published:** 2020-10-13

**Authors:** Lorcan O'Neill, Maria Rodrigues da Costa, Finola Leonard, James Gibbons, Julia Adriana Calderón Díaz, Gerard McCutcheon, Edgar García Manzanilla

**Affiliations:** ^1^Pig Development Department, Teagasc, The Irish Food and Agriculture Authority, Moorepark, Fermoy, Ireland; ^2^School of Veterinary Medicine, University College Dublin, Dublin, Ireland; ^3^Irish Equine Centre, Naas, Ireland; ^4^Pig Development Department, Teagasc, The Irish Food and Agriculture Authority, Oakpark, Carlow, Ireland

**Keywords:** antimicrobial use, indicators, benchmark, pigs, defined daily dose

## Abstract

The need to reduce antimicrobial use (AMU) in livestock production has led to the establishment of national AMU data collection systems in several countries. However, there is currently no consensus on which AMU indicator should be used and many of the systems have defined their own indicators. This study sought to explore the effect of using different internationally recognized indicators on AMU data collected from Irish pig farms and to determine if they influenced the ranking of farms in a benchmarking system. AMU data for 2016 was collected from 67 pig farms (c. 35% of Irish pig production). Benchmarks were defined using seven AMU indicators: two based on weight of active ingredient; four based on the defined daily doses (DDD) used by the European Medicines Agency and the national monitoring systems of Denmark and the Netherlands; and one based on the treatment incidence (TI200) used in several published studies. An arbitrary “action zone,” characterized by farms above an acceptable level of AMU, was set to the upper quartile (i.e., the top 25% of users, *n* = 17). Each pair of indicators was compared by calculating the Spearman rank correlation and assessing if farms above the threshold for one indicator were also above it for the comparison indicator. The action zone was broadly conserved across all indicators; even when using weight-based indicators. The lowest correlation between indicators was 0.94. Fifteen farms were above the action threshold for at least 6 of the 7 indicators while 10 farms were above the threshold for all indicators. However, there were important differences noted for individual farms between most pairs of indicators. The biggest discrepancies were seen when comparing the TI200 to the weight-based indicators and the TI200 to the DDDA_NED_ (as used by Dutch AMU monitoring system). Indicators using the same numerator were the most similar. All indicators used in this study identified the majority of high users. However, the discrepancies noted highlight the fact that different methods of measuring AMU can affect a benchmarking system. Therefore, careful consideration should be given to the limitations of any indicator chosen for use in an AMU monitoring system.

## Introduction

Antimicrobial resistance (AMR) is a public health issue of global importance (1). There are concerns that antimicrobial use (AMU) in animals plays a role in the emergence and dissemination of AMR bacteria (2, 3). Antimicrobial resistance is frequently detected in zoonotic and commensal bacteria (4) and has been associated with the use of antimicrobials in animals (5, 6). This has led to a concerted effort in many countries to reduce AMU in livestock production (7, 8). Systems to measure, benchmark and monitor AMU in livestock production are considered key components of these efforts and forthcoming European Union (EU) legislation requires all Member States to collect AMU data for the pig, poultry and veal production sectors by 2024 and for all species by 2030 (9). Several AMU data collection systems have already been established in various European countries (10). The longest established and best known of these are Vetstat, which is operated by the Danish Veterinary and Food Administration in Denmark (11); and the sector specific databases overseen by the Netherlands Veterinary Medicines Institute (SDa) in the Netherlands (12). Aggregated veterinary antimicrobial sales data for all species are collected by the European Medicines Agency (EMA) for the European Surveillance of Veterinary Antimicrobial Compounds (ESVAC) project and reported annually (13). Quantification of AMU allows for a comparison of consumption between farms, veterinarians, species, types of production and even countries (14). These data can be used in a benchmarking system whereby end users can compare their performance to their peers and authorities can identify and focus on “high users” for intervention or sanction. Many of the AMU data collection systems in operation allow for benchmarking (10). Notable examples of these include the “yellow card” scheme in Denmark (15) and the “action threshold” for farms and veterinarians in the Netherlands (12, 16) where high users may be subject to increased inspection and restricted access to antimicrobials (17, 18). Quantification of AMU also allows for the monitoring of trends over time, assessment of the impact of interventions to reduce AMU (14) and can provide data to assess the relationship between consumption and the occurrence of AMR (5, 19)

One of the most important considerations when quantifying AMU is the unit of measurement, known as an indicator. Collineau et al. defined such indicators as “the number of ‘technical’ units of measurement (i.e., the amount of antimicrobials) consumed and normalized by the population at risk of being treated in a defined period” (14). The numerator, the amount of antimicrobials consumed, is generally expressed in terms of the weight of active ingredient or the number of Defined Daily Doses (DDD). The DDD system, developed by the World Health organization as a standardized method to measure drug consumption, assigns a specific dose to each drug or product and thus accounts for differences in potency between the various antimicrobial drugs (20). This method was first adopted for use in animals by the Vetstat system (21). Alternatively, the numerator may be expressed in terms of the number of animals treated (or equivalently, the number of treatment days). The denominator is a measure of the population of animals at risk of treatment and can be expressed in terms of the number of individuals in the population or its weight of biomass. The denominator may measure the population in terms of the numbers of animals produced, the numbers of animals present or in terms of animal time (e.g., animal days). The population's weight of biomass is determined by assigning an average weight to its constituent species and, where applicable, production categories or age groups. It is also worth noting, that when considering the particular time period under study (e.g., a calendar year), a certain proportion of the population may have been treated with antimicrobials in the preceding period and furthermore, species with more than one production cycle per year (e.g., pigs and poultry) may not have been at risk for the entire period used to calculate the denominator. Therefore, unless the population is studied batch by batch, measurement of AMU is often a proxy representation of AMU at population level rather than a measurement of actual exposure for every individual/batch.

The benchmarking systems of a selection of European countries have been reviewed by others (22). The various AMU data collection systems may differ, for example, in how they define their DDD lists and/or in the weights they assign to species or production categories (14, 22). Therefore, there are now several AMU indicators in use with none having universal acceptance (22, 23). How an indicator influences farm ranking in a benchmarking system matters because farms may be above a threshold for acceptable use with one indicator but below it if a different one is used. Some studies have shown that the use of different indicators affects the interpretation of AMU at national level (24, 25) and farm level (26–28) but did not assess how these differences would affect a benchmarking system. A few studies have shown that indicators can influence the farm classification in a benchmarking system: for cattle in the UK (29); suckling pigs, finisher pigs and poultry in Germany (30); and poultry in France (31). In pigs, the studies to date have focused on comparing national indicators to those based on ESVAC methodology (27, 30); a wider comparison of currently available indicators is lacking. Furthermore, since these comparisons were limited to specific age groups (27, 28, 30), metrics to benchmark AMU amongst farrow-to-finish farms have not yet been evaluated. The objective of this study was to determine if the use of different indicators to benchmark antimicrobial use affected farm ranking amongst a sample population of Irish farrow-to-finish pig farms. The indicators chosen for evaluation are based on those used by ESVAC for the reporting of antimicrobial sales in the EU, the Danish Integrated Antimicrobial Resistance Monitoring and Research Programme (DANMAP)^1^, the Monitoring of Antimicrobial Resistance and Antibiotic Usage in Animals in the Netherlands (MARAN)^2^ and SDa^3^ reports in the Netherlands as well as an indicator developed for use in several international studies (32–34).

## Methods

### Data Collection

Antimicrobial use data for the 2016 calendar year were collected from farrow-to-finish pig farms in Ireland as part of cross-sectional study investigating AMU. Details of the data collection and descriptive results are reported elsewhere (35). Briefly, all 107 client farms of the Teagasc^4^ Pig Development Department advisory service were invited to enroll in the study; 67 volunteered to participate. The sampled farms had a combined sow population of 48,000 and thus represented ~35% of the Irish national herd in that year (36). Farm visits were conducted between September 2017 and September 2018. Farmers provided details about their antimicrobial use in medicated feed, namely, the diets and age groups treated along with the antimicrobials used. Prescription and or invoice records were consulted to determine the numbers of injectable antimicrobial preparations and oral remedies (not for premix) that were used. The farms also submit quarterly performance and production data to the e-Profit Monitor (ePM) database operated by Teagasc and this was consulted to extract population data and feed consumption data (to calculate amounts of medicated feed used) for each participating farm. Eight farms did not submit data to the ePM and provided the relevant production data directly. Further details of the data collection and quantification of antimicrobial use can be found in Appendix A in Supplementary Materials.

### Calculation of Antimicrobial Use Indicators

Using the data collected, AMU for each farm was calculated using seven different indicators. The AMU indicators chosen for comparison in this study are presented in Table 1. This table also presents further information on the development and usage of these indicators.

**Table 1:** Summary explanation of the antimicrobial use indicators used in the study.

**Indicator**	**Developed by**	**Numerator**	**Denominator**	**Comments**
mg/kg lwt milligram per kilogram liveweight sold	Generic indicator	Weight of active ingredient	Liveweight of animals sent to slaughter or sold from farm	
mg/PCU milligram per population correction unit	EMA - ESVAC for reporting of antimicrobial sales in EU/EEA (37)	Weight of active ingredient	PCU; uses numbers of living sows and animals sold from the farm (e.g., for slaughter) Assigned weights: weaners 25 kg; finishers, 65 kg; sows, 240 kg (37)	The PCU was designed for use at national level using census, slaughter and, export/import data (37). These principles are adapted to farm level for this study.
DDD_vet_/PCU defined daily dose per population correction unit	EMA - proposed for use when AMU data stratified by species is available (37, 38)	Treatable kilograms (TK_DDDvet_): Defined doses based on DDD_vet_ for pigs (39)	PCU; see mg/PCU above	Not currently in use for ESVAC reports. Included in SDa national report for the AMU in the Netherlands in 2016 as a comparison to DDDA_NAT_ (40)
DAPD proportion of animal population in treatment per day (expressed per 1000 animals)	DANMAP - for reporting of AMU in Denmark (41)	Treatable kilograms (TK_DEN_): Defined doses based on DADD values (42)	Biomass days; Uses the numbers of animals produced. Assigned weights: piglets 4 kg; weaners (<30 kg), 18.5 kg; finishers (>30 kg), 68.5 kg; sows, 200 kg	DANMAP defines average weights and length of stay in each age group to calculate biomass days. These parameters are based on national performance data. The performance data from the sample farms was used in the same way (41).
DDDA_NED_ defined daily dose animal in the Netherlands	Netherlands Veterinary Medicines Institute (SDa) - for reporting of AMU in the Netherlands (40, 43)	Treatable kilograms (TK_NED_): Defined doses based on product level values in the DG Standard veterinary medicines database (44)	Animal year (AY); the denominator used by SDa in the Netherlands (43) Uses the average numbers of animals present (or the number of animal places) Assigned weights: piglets (<20 kg), 10 kg; finishers, 70 kg; other pigs, 70.2 kg; sows, 220 kg	The DDDA_NED_ is equivalent to the DDDA_NAT_ used to report AMU at national level in the Netherlands (43). It is renamed to reflect its use in this study at farm level.
DDD_vet_/AY defined daily dose per animal year	SDa (40)	TK_DDDvet_	Animal year (AY); see DDDA_NED_	Included in Dutch national reports since 2016 along with DDDA_NAT_ (40)
TI200 treatment incidence (for 200-day lifespan)	Defined for use in pigs by Timmermann et al. (32). Adapted by Sjolund et al. (33) and Sarrazin et al. (34)	Number of animal treatment days using DDD_vet_ as defined dose and standard weights at treatment: piglets 4 kg; weaners 12 kg, finishers 50 kg (34, 45)	Number of animal days in the rearing period (birth to slaughter)	Recalculates the combined TIs for piglets, weaners and finishers into the TI200 for a standardized 200-day lifespan as per Sarrazin et al. (34)

*AMU, antimicrobial use; DANMAP, Danish Integrated Antimicrobial Resistance Monitoring and Research Programme; DADD, defined animal daily doses, the DDD system used by DANMAP; DDD, Defined Daily Dose; DDD_vet_, the DDD system developed for ESVAC; DDDA_NAT_, defined daily dose animals, the indicator used to report AMU at national level in the Netherlands; EMA, European Medicines Agency; ESVAC, European Surveillance of Veterinary Antimicrobial Consumption; EU, European Union; EEA, European Economic Area; PCU, population correction unit; SDa, Netherlands Veterinary Medicines Institute; TI, treatment incidence; TK, treatable kilograms*.

In general, an indicator of AMU can be expressed as follows:

$$\begin{array}{l} \text{indicator }=\frac{\text{numerator}}{\text{denominator}} \end{array}$$

The numerators and denominators for each indicator were calculated using the general principles outlined below and with the appropriate DDDs and assigned weights.

The AMU indicators used at farm level in Denmark, the ADD (animal daily dose), and in the Netherlands, the DDDA_F_ (defined daily dose animal, farm), are stage specific (11, 12). Since the aim of this study was to explore the use of indicators to measure AMU on farrow-to finish farms, the methods used to measure AMU in the pig population at national level in both countries were applied at farm level instead of using the stage specific metrics. For Denmark, this is the DAPD (proportion of animal population in treatment per day) (41) and in the Netherlands it is the DDDA_NAT_ (defined daily animal dose in the Netherlands) (43). Therefore, the metric which uses the DDDA_NAT_ methodology at farm level in this study is termed the DDDA_NED_ in order to avoid confusion with the DDDA_NAT_ and the DDDA_F_.

#### Numerator

Firstly, for each farm, the amounts of active ingredient in each antimicrobial product used were calculated according the protocols outlined by the EMA (46). For the weight-based indicators, i.e., milligram per population correction unit (mg/PCU) and milligram per kilogram liveweight sold (mg/kg lwt), the numerator for an individual farm was simply the sum of the weights of each active ingredient used.

To determine the numerator for the DDD-based indicators the amount of active ingredient for each antimicrobial was converted to “treatable kilograms.” In this study, treatable kilograms (TK) represents the number of kilograms of pig that can be treated with a given amount of antimicrobial if a defined dose is used. It is based on the definition outlined by the Netherlands Veterinary Medicines Institute (40).

(1) $$\begin{array}{r} {\text{TK}}_{\text{DDD}}=\frac{\text{weight of active ingredient}}{\text{DDD}\left(\text{mg}/\text{kg}\right)} \end{array}$$

For the DDD_vet_/PCU and DDD_vet_/AY indicators, the treatable kilograms (TK_DDDvet_) for each antimicrobial were calculated using the DDD_vet_ list for pigs (39). For the antimicrobials with no assigned DDD_vet_ (tulathromycin and tildipirosin) the consensus DDDs defined by Postma et al. were used and adjusted for duration of action using long acting factors (47). The treatable kilograms (TK_DEN_) for the DAPD indicator used the Defined Animal Daily Doses (DADD) applied in the Danish Integrated Antimicrobial Resistance Monitoring and Research Programme (DANMAP) reports (42). Finally, for the DDDA_NED_ indicator, the treatable kilograms (TK_NED_) were calculated using the DG standard available on the SDa website (44). For each indicator, the total TK for each farm was the sum of the TKs for each antimicrobial used.

Each DDD system employs different methodologies. The DDD_vet_ is defined for each antimicrobial by species and route of administration based on the average of doses obtained from the SPC documents from nine European countries (48). The Danish equivalent, the DADD (used to calculate the DAPD), is based on approved doses for each antimicrobial, route of administration, pharmaceutical form and species (42). Dutch DDDA values are defined for each product based on the SPC document, meaning that identical antimicrobial preparations can have different DDDAs (44). The DDD systems also differ in how they treat combination products such as potentiated sulphonamides; in the Netherlands they are considered as one treatment (40) whereas the DDD_vet_ and Danish DADD treats each antimicrobial separately (39, 42).

#### Denominator

The denominator represents the population of animals at risk of treatment. Each denominator partitions the pig population into age group or production categories and assigns each one a standard weight. For example, the PCU assigns finisher pigs a weight of 65 kg (see Table 1). The weight of biomass in each category is calculated by multiplying the numbers of animals by the assigned weight and the total denominator is simply the sum of all the weights. A detailed description of the calculation of the denominators is available in Appendix B in Supplementary Materials.

#### Treatment Incidence

Treatment incidence, first defined by Timmerman et al. describes the percentage of pigs in a stage of production treated with a dose of antimicrobial each day or, equivalently, the percentage time of the period at risk for which a pig was treated (32). The TI indicator, as adapted by Sarrazin et al. (34), is calculated as follows:

$$\begin{array}{l} \text{TI}=\frac{\text{amount of antimicrobial used}\left(\text{mg}\right)}{\text{DDDvet }\left(\text{mg}/\text{kg}\right)\times \#\text{ animals at risk }\times \text{ assigned weight }\left(\text{kg}\right)\times \text{ number of days at risk}}\times 100\text{ animals at risk} \end{array}$$

The TI, which is based on the DDD_vet_, was calculated separately for piglets, weaners and finishers using respective assigned weights of 4, 12, and 50 kg (45). The number of animals and the length of stay in each section were extracted from the ePM or provided by the farmer directly. The TIs were combined and recalculated as the TI200, representing a standardized 200-day lifespan, using the formula defined by Sjölund et al. (33):

$$\begin{array}{l} {\text{TI}200}_{\;}=\frac{\text{TIpiglet }\times \text{suckling period}+\text{TIweaner}\times \text{weaner period }+\text{ TIfinisher}\times \text{finishing period}}{\text{total rearing period}}\;\times \frac{200\;\left(\text{standard life span}\right)}{\text{total rearing period}} \end{array}$$

Assigning a standard weight to each stage means that the weight at the time of treatment is accounted for (albeit based on an estimated standard) and allows for an estimation of the numbers of animals treated. Therefore, in contrast to the other dose-based indicators which consider only the numbers of kilograms treated, the numerator for the TI200 equates to the number of animal treatment days.

### Data Processing and Statistical Analysis

All data were entered into a Microsoft® Excel 365 spreadsheet. Calculations of indicators and statistical analysis were carried out using Microsoft® Excel and R version 3.4.2 (49). Data visualizations was carried out in R using the ggplot2 package (50).

Spearman rank correlations were determined for each pair of indicators. An arbitrary threshold to define excessive AMU was set to the upper quartile (*n* = 17) for each indicator. Farms above this threshold were defined as being in the “action zone” whereby they could theoretically be targeted for intervention to reduce AMU. For each pair of indicators, the number of farms above the threshold for one of the indicators but below for the other was determined. Kappa coefficients were calculated for each pair of indicators to assess the overall agreement between benchmarking classifications (i.e., in action zone or not). The kappa coefficient measures the agreement between rating methods and ranges from 1 (perfect agreement) to <0 (51). Finally, for each pairwise comparison the change in rank for every farm between the two indicators was calculated.

The above pairwise analysis was repeated for injectable antimicrobials from the same AMU dataset. This was done to explore the effect of the indicators on a dataset with a different antimicrobial use profile.

The effect of selected antimicrobial use practices on farm ranking was assessed by comparing the relative rank between selected pairs of indicators between the farms that engaged in the practice and those that did not. The pairs of AMU indicators and antimicrobial use practices assessed were as follows: (1) DAPD vs. DDD_vet_/PCU for the use of tylosin oral premix in medicated feed; DADD (used to calculate the DAPD) = 4 mg/kg (42), DDD_vet_ = 12 mg/kg (39), (2) DDDA_NED_ vs. DDD_vet_/PCU for the use of trimethoprim and sulfadiazine (TMS) oral premix; combination products such TMS are assigned a single DDD by the SDa (40, 44), separate DDD_vet_ values are assigned to each constituent antimicrobial by the EMA (39), and (3) mg/PCU vs. DDD_vet_/PCU for the use of injectable tulathromycin; DDD_vet_, = 0.36 mg/kg (47). For each farm, the relative rank was calculated by subtracting the farm's rank with the second named indicator from the rank with the first named indicator. The results were visualized using box and scatter plots.

## Results

### Quantification of Antimicrobial Use

**Figure 1:**
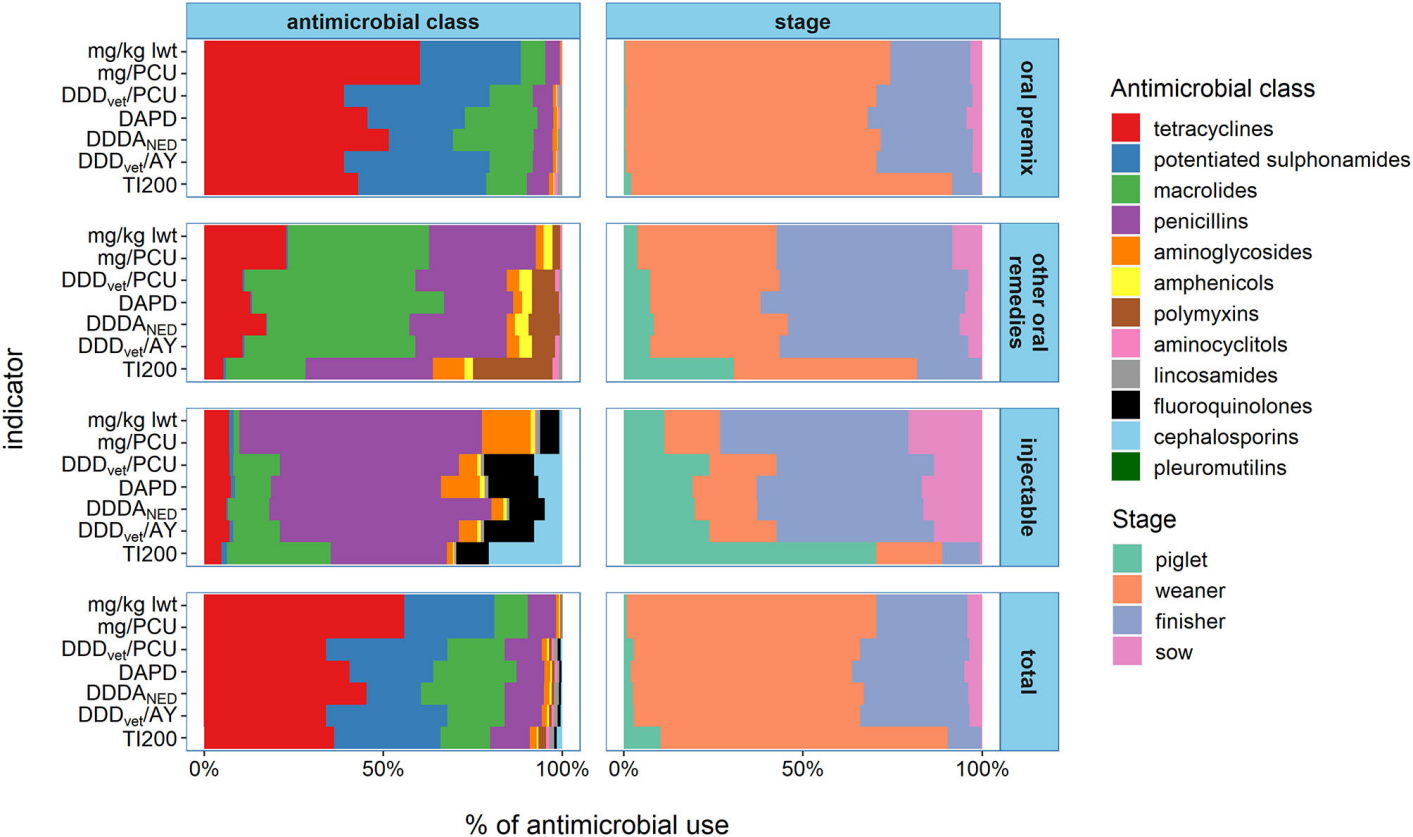
Summary of antimicrobial use for 67 farms in 2016 by antimicrobial class and stage of production measured in the various numerators and stratified by route of administration. Legend: mg/kg lwt, milligram per kilogram liveweight sold; mg/PCU, milligram per population correction unit; DDD_vet_/PCU, defined daily dose per population correction unit; DAPD, proportion of animal population in treatment per day; DDDA_NED_, defined daily dose animal in the Netherlands; DDD_vet_/AY, defined daily dose per animal year; TI200, treatment incidence (TI200). Note that the mg/kg lwt and mg/PCU share the same numerator (weight of active ingredient) as do the DDD_vet_/PCU and DDD_vet_/AY [treatable kilograms (DDD_vet_)].

Table 2 provides a summary of AMU at farm level as measured by each of the indicators and for each route of administration. A detailed description of AMU on the sample farms is reported elsewhere (35). Table 2 also presents the breakdown of AMU for the primary routes of administration in each indicator for the combined sample population. Medicated feed accounted for the majority of AMU and ranged from 82.5 to 89.2% of consumption depending on the indicator used whereas consumption accounted for by injectable antimicrobials ranged from 2.5 to 7.9%. Figure 1 visualizes the breakdown of AMU in each route of administration by antimicrobial class and stage of production.

**Table 2:** Summary of antimicrobial use (AMU) at farm level expressed in various indicators for total AMU (overall), AMU with oral premix and AMU with injectable antimicrobials.

	**Summary statistics for AMU at farm level**			**Breakdown of AMU by route of administration**		
	**Overall**	**Oral premix**	**Injectable**	**Oral premix**	**Other oral remedies**	**Injectable**
mg/kg lwt	63.34 (18.29–153.33)	54.31 (9.72–150.61)	2.79 (1.38–4.01)	89.2%	8.3%	2.5%
mg/PCU	93.93 (25.14–214.64)	78.25 (13.82–205.20)	3.91 (2.07–5.84)	89.2%	8.3%	2.5%
DDD_vet_/PCU	4.50 (1.50–9.97)	3.66 (0.83–8.75)	0.41 (0.25–0.70)	83.1%	10.7%	6.2%
DAPD	40.49 (14.11–92.41)	31.64 (7.16–80.63)	2.84 (1.81–5.32)	85.2%	10.4%	4.4%
DDDA_NED_	11.91 (4.09–28.47)	8.44 (2.18–23.73)	1.18 (0.77–2.25)	84.0%	8.9%	7.1%
DDD_vet_/AY	9.83 (3.49–20.95)	7.59 (1.84–18.26)	0.90 (0.58–1.56)	83.1%	10.7%	6.2%
TI200	15.37 (6.05–35.67)	12.87 (3.41–29.99)	1.17 (0.69–2.15)	82.5%	9.6%	7.9%

*Median values are shown with the interquartile range in brackets. The percentage breakdown of consumption by route of administration is also shown*.

*mg/kg lwt, milligram per kilogram liveweight sold; mg/PCU, milligram per population correction unit; DDD_vet_/PCU, defined daily dose per population correction unit; DAPD, proportion of animal population in treatment per day; DDDA_NED_, defined daily dose animal in the Netherlands; DDD_vet_/AY, defined daily dose per animal year; TI200, treatment incidence (TI200). Note that the mg/kg lwt and mg/PCU share the same numerator (weight of active ingredient) as do the DDD_vet_/PCU and DDD_vet_/AY [treatable kilograms (DDD_vet_)]*.

### Comparison of Indicators

**Figure 2:**
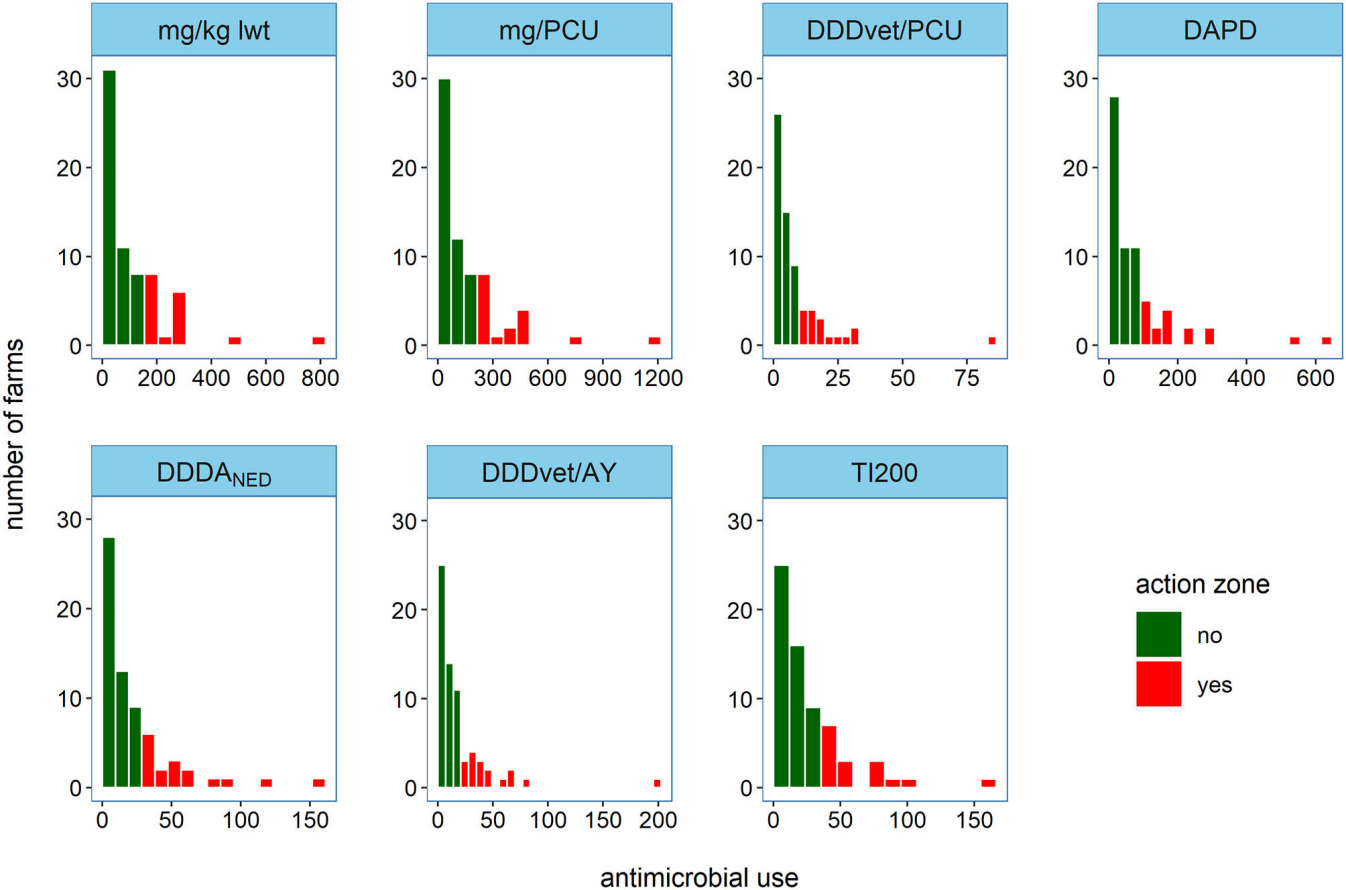
Frequency distribution of antimicrobial use from 67 farms in 2016 measured by each indicator. The action zone was defined as the upper quartile of AMU (*n* = 17). Legend: mg/kg lwt, milligram per kilogram liveweight sold; mg/PCU, milligram per population correction unit; DDD_vet_/PCU, defined daily dose per population correction unit; DAPD, proportion of animal population in treatment per day; DDDA_NED_, defined daily dose animal in the Netherlands; DDD_vet_/AY, defined daily dose per animal year; TI200, treatment incidence (TI200).

**Figure 3:**
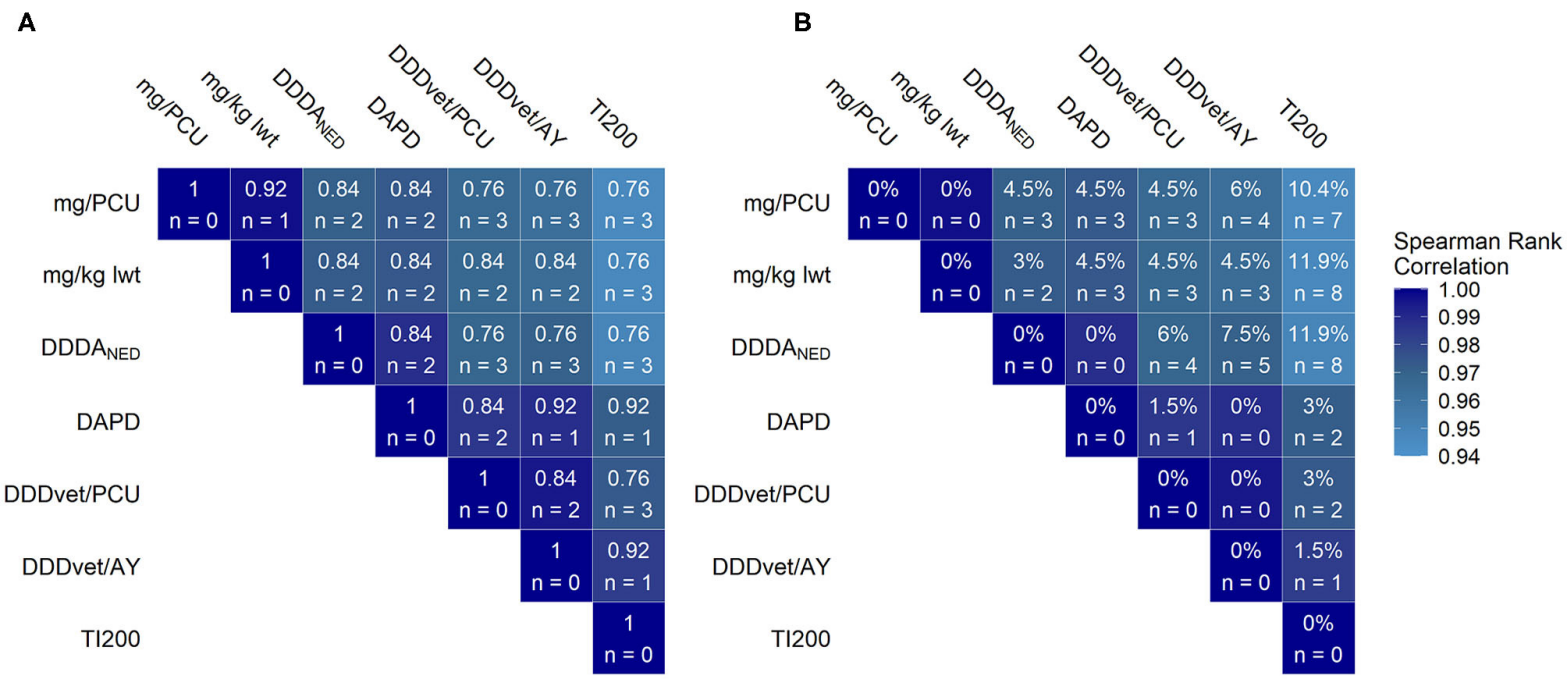
Pairwise comparison of antimicrobial use (AMU) benchmarking systems using the various AMU indicators for all antimicrobial use; 67 farms, 2016. The color of the tile indicates the Spearman's rank correlation coefficient of each pair of indicators. **(A)** The values within the tiles indicate the kappa coefficient and the number of farms ranked in the AMU “action zone” (threshold = upper quartile of AMU) with one indicator but below the threshold in the comparison indicator. **(B)** The values within the tiles indicate the percentage of farms who's rank changed 10 or more places when comparing a given pair of indicators. Legend: mg/PCU, milligram per population correction unit; mg/kg lwt, milligram per kilogram liveweight sold; DDDA_NED_, defined daily dose animal in the Netherlands; DAPD, proportion of animal population in treatment per day; DDD_vet_/PCU, defined daily dose per population correction unit; DDD_vet_/AY, defined daily dose per animal year; TI200, treatment incidence (TI200).

The frequency distributions of AMU for the 67 sample farms measured using the seven indicators is shown in Figure 2. Figure 3 summarizes the pairwise comparison between each of the seven AMU indicators for the complete AMU dataset and shows the Spearman rank correlation coefficients (color code); the number of farms exchanging places between zones and the associated kappa coefficients (Figure 3A); and number of farms moving 10 or more places in rank between each pair (Figure 3B). Overall, 15 farms out of 17 were classified in the action zone for at least six of the indicators while 10 farms out of 17 were above the threshold for all seven.

**Figure 4:**
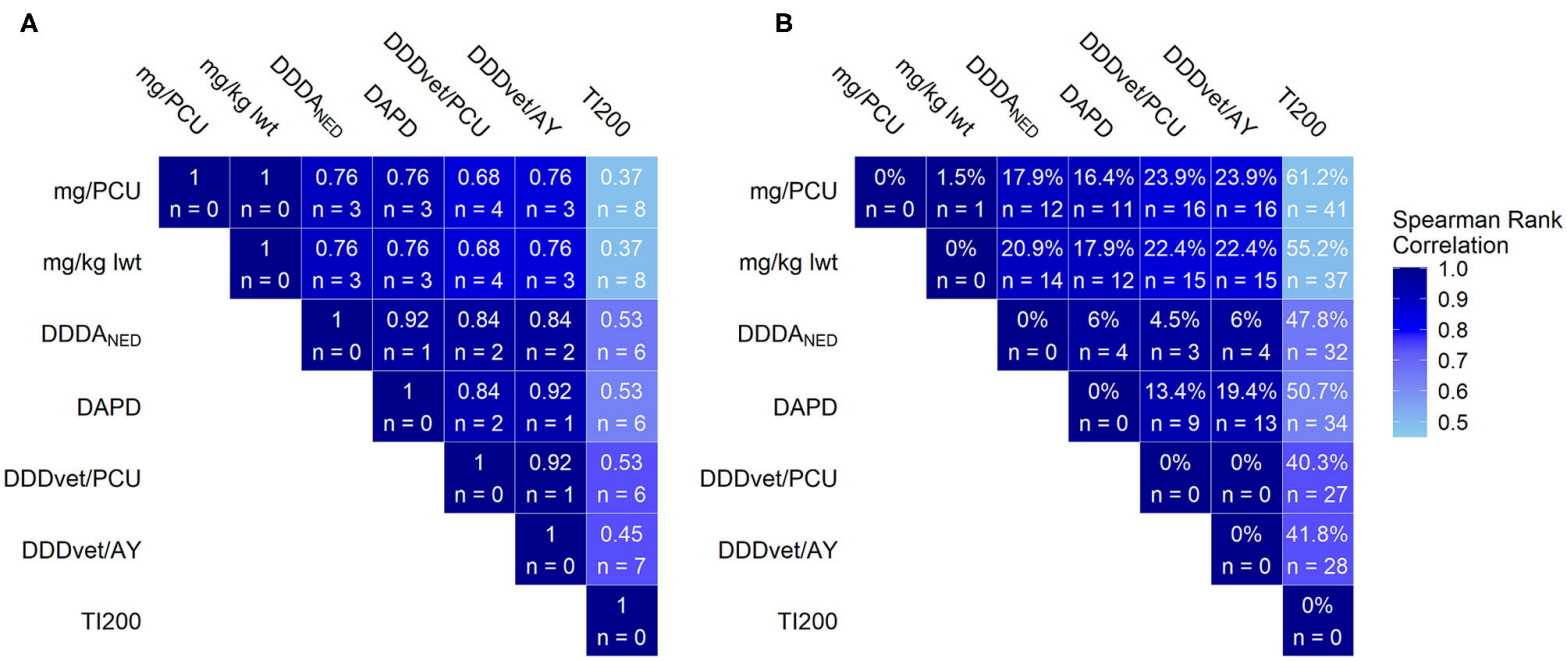
Pairwise comparison of antimicrobial use AMU benchmarking systems using the various AMU indicators for injectable antimicrobial use; 67 farms, 2016. The color of the tile indicates the Spearman's rank correlation coefficient of each pair of indicators. **(A)** The values within the tiles indicate the kappa coefficient and the number of farms ranked in the AMU “action zone” (threshold = upper quartile of AMU) with one indicator but below the threshold in the comparison indicator. **(B)** The values within the tiles indicate the percentage of farms who's rank changed 10 or more places when comparing a given pair of indicators. Legend: mg/PCU, milligram per population correction unit; mg/kg lwt, milligram per kilogram liveweight sold; DDDA_NED_, defined daily dose animal in the Netherlands; DAPD, proportion of animal population in treatment per day; DDD_vet_/PCU, defined daily dose per population correction unit; DDD_vet_/AY, defined daily dose per animal year; TI200, treatment incidence (TI200).

The results of the pairwise comparison of indicators using the injectable AMU dataset are summarized in Figure 4 using the same format outlined for Figure 3 above. Twelve farms out of 17 were in the action zone for at least six of the seven indicators while eight out of 17 were there for all seven.

### Effect of Selected Antimicrobial Use Practices on Farm Ranking

**Figure 5:**
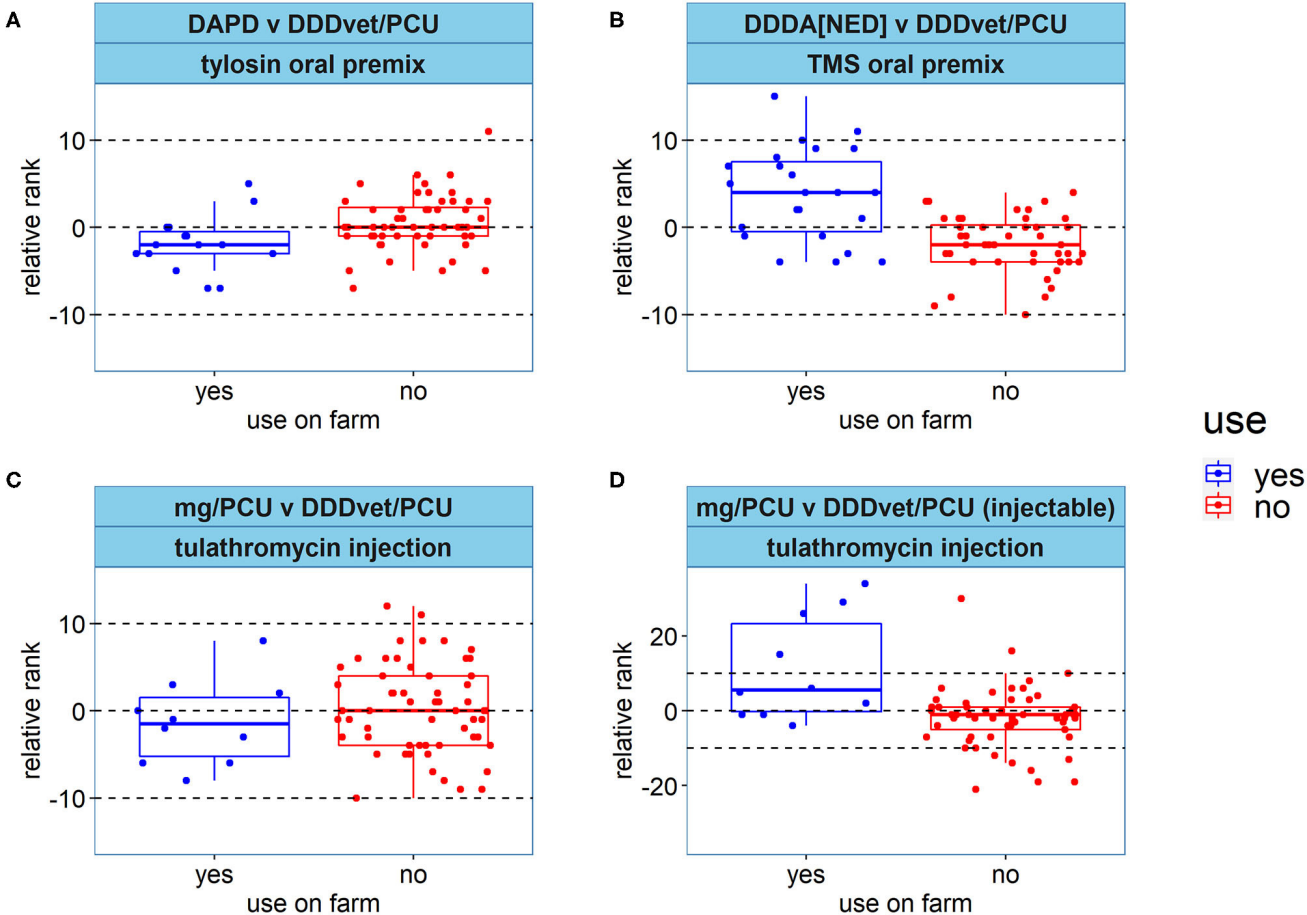
Comparison of farm rank between indicators for selected antimicrobial use (AMU) practices. Positive relative rank values mean the farm ranked higher for the first named indicator; negative relative rank means the farm ranked higher for the second named indicator. **(A–C)** show the comparisons for the complete AMU dataset. **(D)** shows the comparison for the injectable AMU dataset. **(A)** Comparison of relative rank between DAPD and DDD_vet_/PCU for tylosin oral premix. DADD oral premix = 4 mg/kg; the DDD_vet_ = 12 mg/kg. **(B)** Comparison of relative rank between DDDA_NED_ and DDD_vet_/PCU for farms using potentiated sulphonamides in medicated feed. The DDDA_NED_ treats combination products as a single treatment, the DDD_vet_ assigns a DDD to each component separately. **(C,D**) Comparison of relative rank between mg/PCU and DDDvet/PCU for farms using injectable tulathromycin; DDD_vet_ = 0.36 mg/kg. Legend: DAPD, proportion of animal population in treatment per day; DDD_vet_/PCU, defined daily dose per population correction unit; DDDA_NED_, defined daily dose animal in the Netherlands; TMS, trimethoprim and sulfadiazine; mg/PCU, milligram per population correction unit.

The effect of selected antimicrobial use practices on farm ranking is visualized in Figure 5. Eleven out of 15 farms using tylosin oral premix had a lower rank when measured in DDDvet/PCU compared to the DAPD (Figure 5A). Of the 23 farms that used trimethoprim/sulfadiazine oral premix, 16 had a lower rank when measured with the DDDA_NED_ compared to the DDDvet/PCU (Figure 5B). Ten farms used injectable tulathromycin. Seven of those farms ranked lower when AMU was measured in mg/PCU compared to DDDvet/PCU for the injectable AMU dataset (Figure 5D). This effect was not apparent for the complete AMU dataset.

## Discussion

This study explored the effect of using different indicators on a theoretical AMU benchmarking system created for a sample population of 67 Irish pig farms. The study farms represented ~35% of the Irish pig herd and the farrow-to-finish system, operated by all herds, accounts for virtually all pig production in the country (52). The insights gained from this study are likely to be applicable to any future efforts to benchmark AMU amongst pig farms. The indicators chosen for investigation have been used in national and international AMU reports and employ a variety of methods to calculate their respective numerators and denominators. Although each indicator measures the same event, i.e., antimicrobial use on the farm during the year, the outcome is expressed in a different way. The mg/kg lwt and mg/PCU express AMU in terms of mg of active ingredient per kg of animal produced. The DDD based systems, in contrast, express AMU in terms of the weight of biomass treated per kg of animal. Here the interpretation depends on the denominator: it is per kg of animal produced if the PCU denominator is used (DDD_vet_/PCU); per kg animal present (or animal place) per year if the AY is used (DDDA_NAT_ and DDD_vet_/AY); and, per kg biomass day for the DAPD. Finally, the TI200 expresses the percentage of animals in treatment per day (or the percentage of their lifespan spent in treatment) by using standardized weights for each age group to estimate the numbers of animals treated. These disparate measures make comparison of the absolute values obtained from the different indicators challenging and it is further complicated by the different weightings applied to the various antimicrobials and categories of pig. Therefore, the effect on farm ranking in a benchmarking system was used to evaluate the differences between indicators and the central hypothesis of this study was that the different methodologies employed by each indicator would produce different results in terms of whether farms were classified as “high users” or not. The threshold to define an action zone, characterized by farms with unacceptably high AMU, was arbitrarily set to the upper quartile. This method has been used by others (30, 31) and is not intended to reflect what any future threshold should be. In the Netherlands, for example, these thresholds are species and production category specific, and have evolved over time in response to changing patterns of AMU (53).

When applied to the complete Irish AMU dataset, the seven indicators produced similar results. All AMU indicators showed similar right skewed distributions, as reported in other studies in pigs (54), in cattle (55) and in sheep (56), indicating a distinct subset of the population with high AMU. The action zone, which, for the purpose of this study consisted of the 17 farms with the highest AMU, was broadly conserved. For each indicator pair, no more than three farms (4.5% of sample) exchanged places between zones. Fifteen farms were in the action zone for six out of seven indicators while 10 were in all seven. Therefore, while the use of different indicators did affect farm ranking, these fluctuations did not cause widespread changes to the action zones. Echtermann et al. found high correlations between indicators based on Swiss and EMA defined doses and concluded that both systems would produce similar results in a benchmarking system (27). In the present study, relatively high levels of agreement held true even when comparing weight- and dose-based indicators. Another study which applied different indicators to AMU data from poultry had a similar finding, contrary to its authors' expectations, and proposed that low variation in patterns of AMU between farms might explain this unexpected result (31). Routine prophylactic administration of medicated feed to weaned piglets was the predominant AMU practice on Irish pig farms. Four classes of antimicrobials, tetracyclines, potentiated sulphonamides, macrolides and penicillins, accounted for almost all use. Moreover, high use was always associated with medication of older weaner pigs and or finisher pigs (35). Therefore, it seems that the overall pattern of use is a more important determinant of rank than the weighting given to the antimicrobial used. In other words, a farm which medicates large portions of the herd for extended periods will almost always rank higher than a farm which does not regardless of the choice of antimicrobial.

The injectable AMU dataset differed from the complete AMU dataset in terms of the antimicrobial class profile with increased relative importance of the macrolide, fluoroquinolone and cephalosporin classes. Members of these three classes are typically more potent than older classes of antimicrobials such as penicillins or tetracyclines. Therefore, AMU could be underestimated on farms using these antimicrobial classes if weight-based indicators are used. This would be problematic since these classes contain the highest priority critically important antimicrobials (CIA) which are considered as the most important to human health (57). In fact, compared to the analysis for the complete AMU dataset, relatively modest reductions in agreement between benchmarking classifications were apparent if the TI200 was excluded from the analysis of the injectable AMU dataset with at most one extra farm exchanging places between zones for each pair. However, there was marked disagreement between indicators pairs involving the TI200. For these comparisons, Spearman rank correlation coefficients ranged from 0.48 to 0.74 (*p* < 0.001) and the kappa coefficients ranged from 0.37 to 0.53 with between six and eight farms exchanging places between zones. There were also larger fluctuations in rank with up to 62.1% of farms moving 10 or more places (for mg/PCU vs. TI200, see Figure 4). The discrepancies between the TI200 and the other indicators can be explained by differences in the method of calculation. Since the TI200 uses the estimated number of treatment days as the numerator, treatments to piglets, weaners and finishers are treated equally. Therefore, farms with high AMU in piglets ranked higher with the TI200 than they did with the other indicators because of the large number of piglets that can be treated with a relatively small amount of antimicrobial. Conversely, farms with high AMU in finishers would rank lower with the TI200 despite the large amounts of antimicrobials needed to medicate heavier animals. This was important for the injectable AMU dataset because in terms of treatment incidence, piglets were the highest consumers (see Figure 1). A similar indicator, the Treatment Frequency (TF), uses the actual weight at treatment and the actual dose administered to measure AMU in Germany (58). Kasabova et al. also found that body weight at treatment influenced the benchmarking system when comparing TF to the DDD_vet_ based indicator (30). This was not an important consideration for TI200 with the complete AMU dataset as medicated feed in the weaners was still the dominant AMU practice. The observation that highest priority CIAs did not have an impact on overall consumption suggests that consideration should be given to benchmarking these separately.

While it did not affect the benchmarking system as much as expected, using different indicators did affect farm rank. Two pairs of indicators [mg/PCU vs. mg/kg lwt and DDD_vet_/PCU vs. DDD_vet_ per animal year (AY)] shared identical numerators and thus differed only in their denominator. These pairs had the highest correlations, the least fluctuation in rank and generally high agreement in the benchmark classification in both analyses While the different denominators produce different absolute values, they are all related to the underlying structure of the pig population. For example, the DDD_vet_/PCU and DDD_vet_/AY differ roughly by a factor of 2.2 which is close to the number of production cycles per sow per year on a farrow-to-finish farm. In other words, each animal place (AY) produces 2.2 pigs (PCU) per year. Similarly, if the kg biomass days denominator (used by the DAPD) and the AY denominator used the same assigned weights, they would differ by a factor of 365 since the former measures treatment per day and the latter treatment per year. Therefore, the denominator has less influence on ranking than the numerator when applied to a specific animal production sector. This does not hold true if one wants to compare AMU between different sectors with different life cycles. In its comparison of the EMA's methodology to its own, the SDa found that AMU in broiler production was lower than for pigs when measured in DDD_vet_/PCU but higher when measured in DDD_vet_/AY (40). This is because there are more production cycles per year in poultry. In terms of the numerator, the biggest discrepancies are between weight-based and dose-based metrics. For instance, the DDD_vet_ value for chlortetracycline is 30 mg/kg while for ceftiofur it is 0.8 mg/kg (39) which raises concerns that a weight-based metric could encourage the use of some of the highest priority critical antimicrobials. The example of tulathromycin illustrated in Figures 5C,D shows that seven of the ten farms that used it had a more favorable rank when mg/PCU was used to measure their injectable AMU compared to the DDD_vet_/PCU. This effect was not apparent for the complete AMU dataset because of its low relative importance compared to oral antimicrobials. However, discrepancies were also apparent between the indicators using different DDD systems. Figure 1 shows that the different DDD systems produce different consumption patterns even though the underlying data for each is identical. This is in agreement with Taverne et al. who found that AMU in the Dutch pig population appeared lower if measured with the Danish metrics (24) and highlights that international AMU comparisons should be made with caution. At farm level, the example of tylosin, noted by Echtermann et al. was also apparent in this study (27). The DDD_vet_ for oral tylosin is 12 mg/kg (39) whereas the DADD for tylosin oral premix used by DANMAP to calculate DAPD is 4 mg/kg (42). In this instance, using a DDD higher than the dose typically used could encourage its use (see Figure 5A). Similarly, assigning separate DDDs to the constituents of combination products might discourage their use, as seen in Figure 5B. The values assigned to DDD have been shown to influence the choice of antimicrobial. In Denmark, Animal Daily Doses (ADD) were defined at product level until it became apparent that products containing the same antimicrobial but with higher labeled doses than their competitors were being used to manipulate AMU reporting (25). Thereafter, the animal daily doses (ADD) - for use with Vetstat - and the DADD (for use in the DANMAP) were defined at the level of active ingredient (42). More recently, the DVFA modified the “yellow card” system by introducing weighting factors for certain antimicrobials (e.g., 1.2 for tetracycline and 10 for colistin, 3rd and 4th generation cephalosporins and fluoroquinolones) and DANMAP has since reported declines in use of both tetracycline and colistin as a result (59).

The appropriate indicator for use in a surveillance system should be as fair as possible to all participants and should not inadvertently promote one AMU practice over another. To this end it may be preferable if the indicator reflects local conditions regarding the DDD system and assigned weights (27, 29). Accounting for the numbers of individual animals treated produced the most divergent results in the benchmarking classification and, as such, the question of whether to use indicators such as the TI or treatment frequency which focus on the number of animals treated or, indicators that focus on the weight of biomass treated, ultimately depends on which is more important in development of AMR; this requires further study. The TI200 and age group specific indicators require accurate attribution of AMU to the correct age group. This can be challenging when collecting AMU data from pig farms with more than one age group, even for well-established data collection systems (53). It is also important that animals are allocated to the correct age group, an issue that may be complicated by variations in terminology used by different farmers as noted by Kasabova et al. (30). These factors meant that the TI200 indicator was the most challenging to determine as its calculation required more detailed knowledge of the population structure on the farm and the length of stay in each section as well as accurate attribution of antimicrobials. The other indicators, on the other hand, required only the amounts of antimicrobials used and basic population data for their calculation. While it is no doubt preferable for an AMU database to collect as much data as possible, AMU data collection in the field can be challenging (34) and comprehensive AMU data collection systems take considerable time and resources to set up (14). Some data collection systems rely on data input by the farmer (10, 60) and in this scenario, the need for a user friendly and easily understandable system should be evident. It should also be remembered that benchmarking is a communication tool whose aim is to increase understanding of antimicrobial stewardship amongst its end users, farmers and veterinarians, and ultimately to promote engagement with efforts to reduce AMU. In this regard, it is preferable that the chosen indicator has meaning to the end users, although, which indicator is most understandable to the lay person has yet to be established. This study, rather than demonstrate the ideal indicator, showed that none were perfect and that even those that are considered less than ideal (i.e., weight-based indicators and/or production-based indicators) had an acceptable performance in identifying high users. Further study is needed to confirm that these findings apply to AMU data in other settings. However, they may be applicable in settings where the time and resources needed to set up a comprehensive data collection system are not yet in place and thus encourage the implementation of a basic system which can be refined later.

## Conclusion

This study demonstrated that the use of different indicators to benchmark AMU produced broadly similar results when applied to AMU data collected from Irish pig farms. Overall patterns of use in terms of treatment duration and age groups treated were more important than the combination of numerator and denominator in determining the benchmarking classification. Careful consideration should be given to the choice of indicator to ensure it gives a fair and accurate comparison of AMU amongst participants and does not unintentionally promote unwanted shifts in AMU practices. Indicators based on weight of active ingredient, which are used by some data collection systems, can be used to give a meaningful benchmarking classification provided their limitations are accounted for.

## Data Availability Statement

The raw data supporting the conclusions of this article will be made available by the authors, without undue reservation.

## Author Contributions

LO'N designed the study, performed the analysis, and drafted the manuscript. EM, JG, and FL were responsible for the conceptualisation and design of, and funding acquisition for the cross-sectional study on which this investigation is based. LO'N, MR, JCD, GM, and EM performed the data collection in the field. LO'N, MR, FL, and EM reviewed and edited the manuscript. All authors contributed to the article and approved the submitted version.

## Conflict of Interest

The authors declare that the research was conducted in the absence of any commercial or financial relationships that could be construed as a potential conflict of interest.

## Footnotes

^1^www.danmap.org

^2^www.wur.nl/en/Research-Results/Research-Institutes/Bioveterinary-Research/In-the-spotlight/Antibiotic-resistance-2/MARAN-reports.htm

^3^ www.autoriteitdiergeneesmiddelen.nl

^4^Teagasc, the Agriculture and Food Development Authority. www.teagasc.ie

## Abbreviations

ADD: animal daily dose
AMR: antimicrobial resistance
AMU: antimicrobial use
AY: animal year
CIA: critically important antimicrobial
DADD: defined animal daily dose
DANMAP: Danish Integrated Antimicrobial Resistance Monitoring and Research Programme
DAPD: proportion of animal population in treatment per day
DDD: defined daily dose
DDDA: defined daily dose animal
DVFA: Danish Veterinary and Food Administration
EEA: European Economic Area
EU: European Union
EMA: European Medicines Agency
ESVAC: European Surveillance of Veterinary Antimicrobial Consumption
PCU: population correction unit
SDa: Netherlands Veterinary Medicines Institute
SPC: summary of product characteristics
TI: treatment incidence
TK: treatable kilograms.
