# Supplementary material for: Does the Use of Different Indicators to Benchmark Antimicrobial Use Affect Farm Ranking?
Source: Front Vet Sci. 2020 Oct 13;7:558793. doi: 10.3389/fvets.2020.558793 (PMC7590364; doi:10.3389/fvets.2020.558793)
Supplement: Supplementary file 1 [file Data_Sheet_1.docx]

**Supplementary Materials**

**Appendix A**

**Quantification of Antimicrobial Use**

The antimicrobial use (AMU) data used in this study were collected during a cross-sectional study investigating AMU on Irish pig farms during 2016. All 107 client farms of the Teagasc Pig Development Department advisory service were invited to participate; 67 volunteered to participate. The following description of the quantification of AMU for the 67 farms is adapted from O’Neill *et al.* (1).

*Medicated feed*

Antimicrobial oral premixes are specifically intended for incorporation into medicated feed by licenced feed mills (2) and are thus distinct from other oral remedies which are added to feed or water on the farm. Diets are specific to the stage of production and any of these diets may be medicated with antimicrobials. Farmers were asked to indicate if each diet was medicated during the year, which antimicrobial(s) were used and their inclusion rates. The amount of medicated diet used in each category for each antimicrobial included was determined by 1) consulting data submitted to the Teagasc e-Profit Monitor (ePM) database; 2) invoice records; or 3) data provided directly by the farmer. To calculate the amounts of antimicrobial administered in medicated feed the following formula was used:

$$weight of medicated feed (kg) \times inclusion rate of active ingredient (mg/kg)$$

The inclusion rate was expressed in terms of mg of antimicrobial per kg of medicated feed (e.g. chlortetracycline 300mg/kg). This calculation was performed for every combination of diet and antimicrobial inclusion rate. Since the medicated diets were stage specific, it was possible to attribute all the antimicrobial oral premixes to the correct age group.

*Other oral remedies and injectable preparations*

Farmers provided prescription and or invoice to determine the amounts of antimicrobials used during 2016. Farmers were asked to indicate which age group each antimicrobial was used in. Complete prescription data were not available for five farms and in these cases, missing values for the antimicrobials used on the farm were imputed based on estimates provided by the farmer or the median value of use on the other farms using same product. The amount of antimicrobial used was calculated using the following formula for each preparation of each active ingredient:

$$number of packs\times pack size(g or ml)\times strength (mg/ml or mg/g)$$

It was not possible in every case to determine the amount used in each age group that the product was prescribed for. In such instances, the product was allocated to the relevant age categories in proportion to the weight of biomass of the given age category in accordance with the method used by Sarrazin *et al*. (3).

**References**

1. O’Neill L, Rodrigues da Costa M, Leonard FC, Gibbons J, Calderón Díaz JA, McCutcheon G *et al*. Quantification, description and international comparison of antimicrobial use on Irish pig farms. Porcine Health Management (2020) In press.
2. Regulation (EU) 2019/4 of the European Parliament and of the Council of 11 December 2018 on the manufacture, placing on the market and use of medicated feed, amending Regulation (EC) No 183/2005 of the European Parliament and of the Council and repealing Council Directive 90/167/EEC (Text with EEA relevance). Official Journal of the European Union. 2019; L4:1–23. ELI: <http://data.europa.eu/eli/reg/2019/4/oj>
3. Sarrazin S, Joosten P, Gompel LV, Luiken RECE, Mevius DJ, Wagenaar JA, *et al*. Quantitative and qualitative analysis of antimicrobial usage patterns in 180 selected farrow-to-finish pig farms from nine European countries based on single batch and purchase data. The Journal of antimicrobial chemotherapy. (2019) 74(3):807–16. [doi: 10.1093/jac/dky503](https://app.readcube.com/)

**Appendix B**

**Calculation of denominators**

*Kilogram liveweight sold (kg lwt)*

The kilogram liveweight sold (kg lwt) is a production-based denominator which is calculated using the weight of animals sold from the farm.

$$kg lwt=(\#finishers sent to slaughter \times average liveweight at slaughter (kg))+(\# sows sent to slaughter \times average liveweight at slaughter (kg))$$

The numbers of animals sold and average weights were extracted from the ePM or provided directly by the farmer. The average liveweight at slaughter for sows was not available for most farms. In these instances, an average weight of 240 kg was assumed.

*Population correction unit (PCU)*

The population correction unit was developed by the European Medicines Agency (EMA) for its European Surveillance of Veterinary Antimicrobial Consumption (ESVAC) project (1). It is calculated using the numbers of breeding animals and the numbers of animals slaughtered or exported and is adjusted for animals imported. Each species and production category within species are assigned a specific weight based on the average weight at the time of treatment (1). The PCU at farm level was calculated using the same principles with the distinction that import and export at farm level refer to animals bought or sold.

In the pigs the assigned weights are: weaners, 25 kg; finishers, 65 kg; sows, 240 kg.

$$PCU=(\# weaners sold\times25 kg)+(\# finishers sent to slaugter)+(\# sows present on the farm \times240 kg)-(\# weaners bought \times25 kg)$$

*Biomass days per year*

The DAPD (proportion of animal population in treatment per day) is the AMU indicator used by the Danish Integrated Antimicrobial Resistance Monitoring and Research Programme (DANMAP) (2). It should be noted that this indicator is used to measure AMU for the entire Danish pig population and is different from the stage specific ADD (animal daily dose) used by the Vetstat benchmarking system at farm level (3).

The DAPD uses biomass days per year as the denominator. The biomass days for each farm is the sum of the biomass days for sows, piglets, weaners and finishers. The calculations used to calculate the biomass days for this study are based on the formula developed by Jensen *et al.* for use in DANMAP and other studies (2, 4, 5).

For sows, the number of biomass days is:

$$\# sows \times average body weight (kg) x 365 days$$

The average body weight of 200kg was used as per DANMAP (5).

For piglets, weaners and finishers, the number of biomass days is:

$$(\# animals produced \times(1 +1/2 mortality))\times\# days in section \times average body weight (kg)$$

The average body weight was calculated using the entrance and exit weights and assumes linear growth:

$$average body weight=entrance weight+(exit weight-entrance weight)/2$$

The number of animals produced, number of days in each section and mortality were extracted from the ePM. The entrance and exit weights used were: piglets, 1 kg and 7 kg; weaners, 7 kg and 30 kg; finishers, 30 kg and 107 kg.

*Animal year*

The animal year (AY) is the denominator used by the Netherlands Veterinary Medicines Institute (SDa) for calculation of the DDDA_F_ (defined daily dose animal, farm) and DDDA_NAT_ (defined daily dose animal, national) (6, 7). The animal year is based on the number of animals present on a given day or the number of animal places. For pigs, separate DDDA_F_ for sows and suckling piglets, weaner pigs and fattening pigs (finishers) are used to measure AMU at farm level. Specific weights are assigned for each age group to calculate the AY denominator for each section. The DDDA_NAT_ is used to measure AMU in the national population; it is similar to the DDDA_F_ but the animal year is calculated using census data and uses different assigned weights (7). Since no DDDA_F_ is defined for farrow-to-finish farms, the methodology used to calculate the DDDA_NAT_ animal year for the pig population at national level was applied to farm level to calculate the AY for each farm in this study.

The categories of pigs and their assigned weights for the animal year used to calculate the DDDA_NAT_ are: piglets (< 20 kg), 10 kg; fattening pigs, 70.2 kg; other pigs, 70 kg; sows, 220 kg (7).

$$animal year=\left( \#piglets \times10 kg \right)+\left( \#fattening pigs \times70.2 kg \right)+ \left( \# other pigs \times70 kg \right)+(\# sows \times220 kg)$$

**References**

1. European Medicines Agency. Trends in the sales of veterinary antimicrobial agents in nine European countries. (2011) Available at <https://www.ema.europa.eu/documents/report/trends-sales-veterinary-antimicrobial-agents-nine-european-countries_en.pdf> (accessed on December 2, 2019)
2. DANMAP (Danish Integrated Antimicrobial Resistance Monitoring and Research Programme). DANMAP 2012. Use of antimicrobial agents and occurrence of antimicrobial resistance in bacteria from food animals, food and humans in Denmark. (2013) ISSN 1600-2032. Available at: <https://www.danmap.org/-/media/arkiv/projekt-sites/danmap/danmap-reports/danmap-2012/danmap_2012.pdf?la=en> (accessed on March 3, 2020)
3. Stege H, Bager F, Jacobsen E, Thougaard A. VETSTAT—the Danish system for surveillance of the veterinary use of drugs for production animals. Preventive Veterinary Medicine. (2003) 57(3):105–15. doi: 10.1016/S0167-5877(02)00233-7
4. Jensen VF. The question of the denominator: Estimating the live animal population. In: Proceedings of Quantification, Benchmarking and Stewardship of Veterinary Antimicrobial Usage: First International Conference ;27-28 February 2018; Ghent, Belgium. Available at: <https://aacting.org/first-aacting-conference/> (accessed on December 2, 2019)
5. Jensen VF, Sommer HM, Struve T, Clausen J, Chriél M. Factors associated with usage of antimicrobials in commercial mink (*Neovison vison*) production in Denmark. Prev Vet Med. 2016; 126:170–82.
6. The Netherlands Veterinary Medicines Institute. Usage of Antibiotics in Agricultural Livestock in the Netherlands in 2016. Trends and benchmarking of livestock farms and veterinarians. (2017) Available at: <https://cdn.i-pulse.nl/autoriteitdiergeneesmiddelen/userfiles/Publications/engels-def-rapportage-2016-deel-1-en-2-22-09-2017.pdf> (accessed on August 19, 2019)
7. The Netherlands Veterinary Institute. Standard operating procedure. Calculation of the DDDA for antimicrobials by the SDa for the cattle, veal, pig, broiler, turkey and rabbit farming sectors. (2020) Available at: <http://cdn.i-pulse.nl/autoriteitdiergeneesmiddelen/userfiles/overige%20rapporten/sop-rekensystematiek-website-03032020.pdf> (accessed April 2, 2020)
